# WNT5 Interacts with the Ryk Receptors Doughnut and Derailed to Mediate Muscle Attachment Site Selection in *Drosophila melanogaster*

**DOI:** 10.1371/journal.pone.0032297

**Published:** 2012-03-05

**Authors:** Liza L. Lahaye, Rene R. Wouda, Anja W. M. de Jong, Lee G. Fradkin, Jasprina N. Noordermeer

**Affiliations:** Laboratory of Developmental Neurobiology, Department of Molecular and Cell Biology, Leiden University Medical Center, Leiden, The Netherlands; VIB and KU Leuven, Belgium

## Abstract

In recent years a number of the genes that regulate muscle formation and maintenance in higher organisms have been identified. Studies employing invertebrate and vertebrate model organisms have revealed that many of the genes required for early mesoderm specification are highly conserved throughout evolution. Less is known about the molecules that mediate the steps subsequent to myogenesis, e. g. myotube guidance and attachment to tendon cells. We use the stereotypic pattern of the *Drosophila* embryonic body wall musculature in genetic approaches to identify novel factors required for muscle attachment site selection. Here, we show that *Wnt5* is needed in this process. The lateral transverse muscles frequently overshoot their target attachment sites and stably attach at novel epidermal sites in W*nt5* mutant embryos. Restoration of WNT5 expression in either the muscle or the tendon cell rescues the mutant phenotype. Surprisingly, the novel attachment sites in *Wnt5* mutants frequently do not express the Stripe (SR) protein which has been shown to be required for terminal tendon cell differentiation. A muscle bypass phenotype was previously reported for embryos lacking the WNT5 receptor Derailed (DRL). *drl* and *Wnt5* mutant embryos also exhibit axon path finding errors. DRL belongs to the conserved Ryk receptor tyrosine kinase family which includes two other *Drosophila* orthologs, the Doughnut on 2 (DNT) and Derailed-2 (DRL-2) proteins. We generated a mutant allele of *dnt* and find that *dnt*, but not *Drl-2*, mutant embryos also show a muscle bypass phenotype. Genetic interaction experiments indicate that *drl* and *dnt* act together, likely as WNT5 receptors, to control muscle attachment site selection. These results extend previous findings that at least some of the molecular pathways that guide axons towards their targets are also employed for guidance of muscle fibers to their appropriate attachment sites.

## Introduction

The establishment of the musculature in higher organisms is a multistep process involving myoblast specification and fusion, followed by guidance of the myotubes towards the muscle attachment sites (MAS) (reviewed in [1]). Final differentiation of both the muscle and the attachment sites is initiated when the multinucleated fiber attaches to the tendon cell. Intercellular communication between the myofiber and the tendon cells mediated by secreted or transmembrane proteins is essential to ensure a stable muscle attachment resistant to contraction-induced detachment (reviewed in [2]). Only a few molecules that regulate these different stages of muscle pattern formation have been identified so far, but most characterized factors show a remarkable degree of functional conservation between vertebrates and invertebrates. The *Drosophila* embryonic body wall musculature with its stereotyped pattern and amenability to genetic analysis has been an excellent model to unravel the cellular and molecular mechanisms underlying this process [2], [3], [4], [5], [6], [7].

The *Drosophila* somatic musculature is established into a stereotypical segmentally reiterated pattern during embryonic development. Pattern formation starts at 7.5 hours after egg laying (AEL) and is completed 5.5 hours later when the muscle fibers form stable contacts with the epidermal tendon cells in the insects' exoskeleton (reviewed in [6]). Muscles persist through the larval stages until the pupal stage when they degenerate and are replaced by the adult musculature [8]. Initially, each embryonic somatic muscle fiber is formed by the fusion of a muscle founder cell and a number of fusion-competent myoblasts [9]. The fusion process creates multinucleated myofibers whose two leading edges subsequently migrate towards clusters of tendon cell progenitors in the epidermis [1], [2], [7].

The initial determination of the tendon cell progenitors in *Drosophila* is provided by segment polarity genes such as *wingless* (*wg*) and *hedgehog* that activate the early growth response (Egr)-like transcription factor Stripe (SR) in segmentally-reiterated clusters of epidermal cells [10]. Once SR is activated these cells become tendon cell progenitors and SR expression is both necessary and sufficient to promote muscle migration towards these cells [11], [12], [13]. However, final differentiation of the single selected tendon cell requires direct interaction with a muscle fiber (reviewed in [2]).

Upon muscle attachment, Vein, a neuregulin-like ligand secreted from muscle, accumulates at the muscle-tendon junction to activate the Epidermal Growth Factor pathway only in the tendon cell progenitor that is contacted by the muscle fiber [14]. This signal maintains SR expression and results in the differentiation of the progenitor into a mature tendon cell. The precursor cells that are not contacted by a muscle fiber cease to express SR and do not differentiate into tendon cells. SR, in turn, induces the expression of both the Slit [15] and Leucine-rich tendon-specific proteins [16]. These proteins then act as positive and negative guidance cues, respectively, for the muscle fibers. The final stage of tendon cell determination is defined by the association of αPS2/βPS Integrin (at the muscle tip) with Thrombospondin (TSP; at the extracellular matrix of the tendon cell) mediating the formation of a myotendinous junction at the attachment site [17], [18]. This junction withstands the mechanical forces that occur during larval locomotion.

Several proteins expressed in the muscle or tendon cells have been shown to control muscle guidance and attachment. The Roundabout (ROBO) protein which is expressed in a subset of muscle fibers, acts as the guidance receptor for Slit produced by the tendon cells [15]. ROBO and Slit interactions are also needed for guidance of axons across the *Drosophila* embryonic ventral midline in the central nervous system (CNS) [19], [20]. The Kontiki (KON) protein is expressed on the tips of a subset of growing myotubes and is needed, in a pathway involving the Glutamate receptor binding protein protein, for their guidance to the correct attachment site [21]. The tendon cell-derived ligand of KON remains to be identified.

Another molecule shown to act as a guidance receptor both for axons and myotubes is DRL (reviewed in [22]). It was initially identified in screens for genes required for axon pathfinding in the *Drosophila* embryo [23], [24] and for learning and memory in the adult [25]. It was also shown to be required for the correct tendon cell attachment of a subset of the lateral transverse muscles (LTMs 21–23) [24].

DRL is a member of the conserved transmembrane receptor tyrosine kinase Ryk family [26], [27] which bears an extracellular Wnt-binding WIF domain and an intracellular tyrosine kinase homologous domain. *C. elegans*, zebra fish and mammals have a single Ryk ortholog while three Ryk proteins, DRL, DRL-2 and DNT, are encoded in the fruit fly genome. DRL-2 and DNT share 35% and 60% amino acid identity, respectively, with DRL.

The *Drosophila* Wnt family member WNT5 acts as a ligand for DRL in the nervous system and both genes are required for the proper axon guidance leading to correct formation of the embryonic ventral cord commissures [28], [29]. Wnt proteins are highly conserved secreted molecules that play roles in diverse signaling pathways acting during normal development and are perturbed during oncogenesis [30]. Wnt genes are also important for the development and function of the nervous system throughout the animal kingdom. They have roles in neuronal differentiation, axon extension, axon guidance and neural circuit assembly in both vertebrates and invertebrates (reviewed in [31], [32]).

A number of at least partially distinct Wnt signaling pathways have been uncovered and, of these, the canonical Wnt pathway is most extensively studied (reviewed in [33]). Wnt binding to the Frizzled and LRP co-receptors results in the accumulation of β-catenin in the cytosol and its translocation to the nucleus where it activates TCF/LEF-dependent transcription of target genes. There are also alternative, non-canonical, Wnt pathways, e. g., the Ca^2+^, Planar Cell Polarity (PCP) and Ryk pathways (reviewed in [22], [30], [34], [35]). The least is known about the downstream signaling components of the Wnt pathway that acts via the RYK receptor.

Wnt/Ryk interactions are essential both in *Drosophila* and mammals for normal nervous system development (reviewed in [22], [31]). Ryks have been characterized as “dead”-tyrosine kinases based on the observation that they contain amino acid substitutions on sites in the potential kinase domain that would likely render them inactive as protein kinases [26], [27]. However, it is still unclear whether Ryk's kinase domain might be active under certain conditions [36]. During mammalian brain neurogenesis, Ryk has been reported to be cleaved at a conserved sequence in the transmembrane domain resulting in the translocation of the cytoplasmic domain to the nucleus where it may regulate transcription [37]. WNT5/DRL signaling during formation of the *Drosophila* embryonic nervous system requires the Src family non-receptor tyrosine kinase, SRC64B [38], indicating that it may be involved in actively transducing an intracellular signal upon WNT5 binding to DRL.

Here, we present evidence that *Wnt5* is required for appropriate attachment of a subset of embryonic muscles, the LTMs 21, 22 and 23. In animals lacking WNT5, myotubes overshoot their normal attachment sites and form ectopic contacts. This bypass phenotype was previously observed in *drl* mutant embryos that lack DRL which is normally expressed in the LTMs [39], [40]. DRL was found to be required in the muscle fiber to rescue the guidance defect [39]. We find that WNT5, a secreted protein, expressed in either the tendon cells or the muscle fiber is sufficient to restore correct muscle attachment in *Wnt5* mutant embryos. The majority of the novel ectopic attachment sites in *drl* and *Wnt5* mutants do not express SR, indicating that it is not needed to form or maintain these novel muscle attachments to the body wall. The ectopic attachment sites persist through larval stages and accumulate Fasciculin2 (FAS2), a cell adhesion protein that is normally present in both the muscle and the tendon cells at the myotendinous junction. Finally, we generated a mutant allele of *dnt*, and found that it is also required for correct LTM attachment, while the third *Drosophila* Ryk family member, *Drl-2*, is not.

## Results

### The embryonic muscles 21 through 23 often overshoot their attachment sites when WNT5 is absent

**Figure 1:**
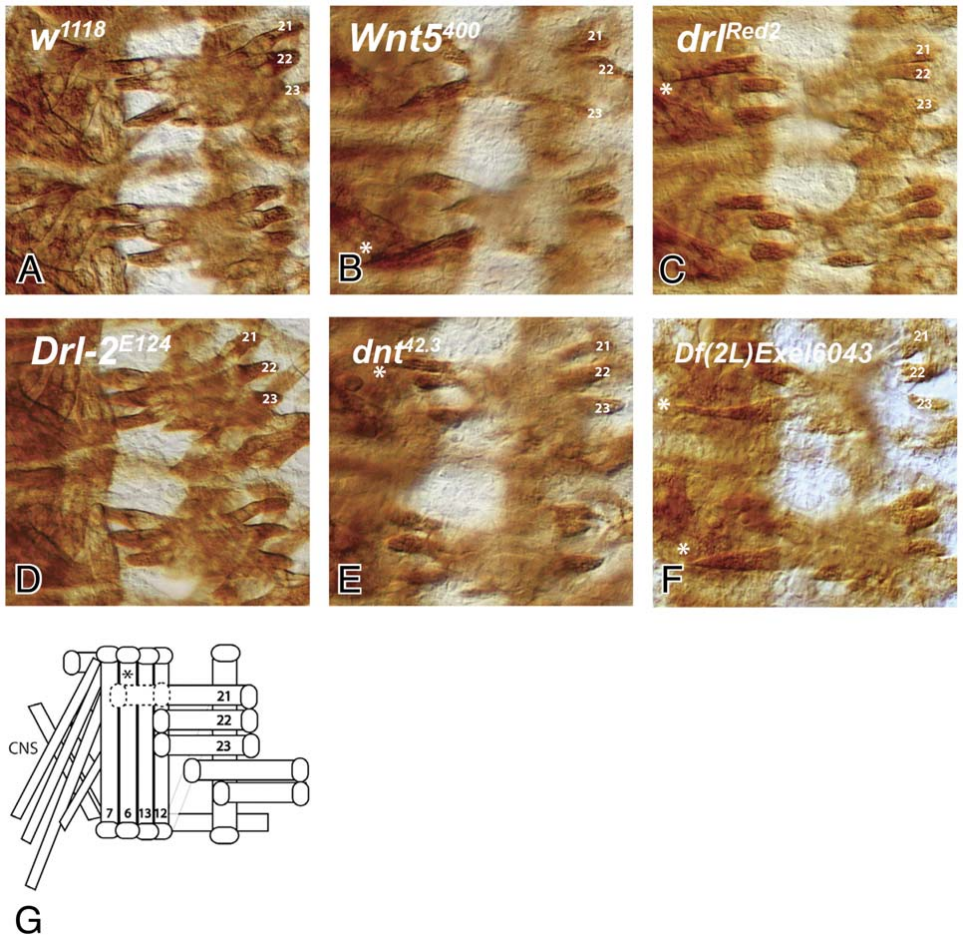
LTM muscle fibers 21, 22 and 23 frequently overshoot their attachment sites in *Wnt5*, *drl* and *dnt* mutant embryos. Stage 16 embryo body wall muscle preparations stained with anti-Muscle Myosin are shown for the wild type control (*w^1118^*) (**A**), *Wnt5^400^* (**B**), *drl^Red2^* (**C**), *Drl-2^E124^* (**D**), *dnt^42.3^* (**E**) and Df(2L)Exel6043 (**F**). Two hemisegments are displayed for each genotype with one set of muscles 21–23 labelled. In *Wnt5*, *drl* and *dnt* mutants, LTMs frequently bypass their normal attachment at the epidermis at muscle 12 and instead extend ventrally beyond muscle 13 and attach at a novel epidermal site located close to muscle fiber 7. Df(2L)Exel6043 mutant embryos, that lack both DNT and DRL, display this phenotype in all hemisegments of the homozygous animals. The penetrance of these phenotypes is shown in **Table 1**. The muscle bypass phenotype is schematically shown in panel (**G**). The * indicates the location of the novel, ectopic epidermal attachment in panels (**B**), (**C**), (**E**), (**F**) and (**G**). Anterior is up and ventral is left.

The somatic mesoderm gives rise to a stereotypic segmentally-reiterated set of body wall muscles during *Drosophila* embryonic development. The muscle pattern of the abdominal hemisegments A2 to A6 consists of 30 fibers that attach at both sides to tendon cells in the epidermis [6]. The DRL receptor is required for the correct attachment site selection by a subset of these muscles, the LTMs (muscles 21–23 [39]). In wild type embryos, the LTMs extend ventrally towards the dorsal border of muscle fiber 12 at which site they attach to a tendon cell in the epidermis (**Figs. 1A and 1G**). However, in the *drl* mutant, the LTMs frequently overshoot their target and extend further ventrally passed muscle 13 or 6 to muscle fiber 7 and attach to an ectopic epidermal attachment site ([39]; **Figs. 1C and 1G**). Usually, only one of the three muscles per hemisegment exhibits this phenotype.

DRL also serves as an axon guidance receptor for the WNT5 protein during embryonic ventral nerve cord commissure formation [29]. We wondered whether *Wnt5* possibly also acts during formation of the embryonic musculature. We therefore examined the morphology of the embryonic muscle pattern in *Wnt5* mutants using an antibody against the Muscle Myosin protein which labels all somatic muscles (Materials and Methods). Most muscles develop normally and attach at their correct position to the body wall in *Wnt5^400^* null mutant embryos. However, one or more of the muscle fibers 21, 22 and 23 extends far more ventrally than in the wild type to attach at the epidermis beyond the ventral border of muscle 13 in 17% of the hemisegments scored (**Fig. 1B** **and** **Table 1**). Occasionally, either muscle 6 or muscle 7 is absent or not correctly attached in these mutants, but we did not observe a correlation between the absence of muscles 6 or 7 and the bypass phenotypes of the LTMs. In the *drl* mutant, this bypass phenotype is more penetrant and is observed in 36% of the hemisegments, while this phenotype was never observed in wild type control embryos (**Fig. 1C** **and** **Table 1**; [39]). Since, the degree of myotube overextension varies somewhat; we scored a fiber as overshooting its target only when it extended ventrally and attached ectopically beyond muscle 13 at the end of embryogenesis (stage 17, 18 hours AEL).

**Table 1:** LTM muscle bypass phenotype in *Wnt5*, *drl* and *dnt* mutants and restoration of attachment when Wnt5 is present in tendon cells or muscle.

Genotype	% hemisegments with bypassing LTM muscles	n = number of hemisegments counted
*w^1118^*	0%	464
*Wnt5^400^*	17%	330
*Wnt5^400^*; UAS-*Wnt5*/+	12%	392
*Wnt5^400^*; 24B-GAL4/+	11%	395
*Wnt5^400^*; UAS-*Wnt5*/24B-GAL4	0%	391
*Wnt5^400^*; mef2-GAL4/+	10%	375
*Wnt5^400^*; UAS-*Wnt5*/mef2-GAL4	0%	334
*Wnt5^400^*; sr-GAL4/+	20%	365
*Wnt5^400^*; UAS-*Wnt5*/sr-GAL4	2%	394
*Wnt5^400^*; UAS-*Wnt5*/P{GawB}tey^5053A^	13%	97
*drl^Red2^*	36%	363
*Drl-2^E124^*	0%	185
*drl^Red2^,Drl-2^E124^*	35%	322
*Wnt5^400^/+; drl^Red2^/+*	0%	200
*dnt^42.3^*	8%	363
*drl^Red2^*/Df(2L)Exel6043	50%	355
*drl^Red2^*/Df(2L)ED1231	50%	354
*dnt^42.3^*/Df(2L)Exel6043	8%	385
Df(2L)ED1231 (*drl*, *dnt*)	94%	49
Df(2L)ED1231/+	0%	100
Df(2L)Exel6043 (*drl*, *dnt*)	96%	183
Df(2L)Exel6043/+	0%	100
*Wnt5^400^*;*drl* ^Red2^	36%	382
*Wnt5^400^*; Df(2L)Exel6043/+	27%	185
*Wnt5^400^*/+; Df(2L)Exel6043/+	16%	95
*Wnt2^L^,Wnt4^C1^*	0%	190
*Wnt5^400^;Wnt2^L^,Wnt4^C1^*	21%	374
*Wnt5^400^* 3^rd^ instar larvae	8%	192
*drl^Red2^* 3^rd^ instar larvae	16%	192

Embryos were stained with anti-Muscle Myosin and hemisegments A2 to A6 were scored for possible bypass by the LTMs. A muscle was scored as bypassing its attachment site when it extended its tip ventrally beyond muscle fiber 13. Embryos were sexed by use of anti-Sex-Lethal where appropriate.

The number of LTMs that bypass their normal attachment sites when *drl* alone or when *drl* and *Wnt5* are both absent are the same (**Table 1**), suggesting that *drl* and *Wnt5* are in one pathway controlling attachment site selection of the LTMs. However, the significant difference in the numbers of overextended LTMs in *Wnt5* mutants (17%) versus that of *drl* mutants (36%) indicates that DRL may bind multiple ligands to mediate muscle guidance. We therefore investigated whether two other *Drosophila* Wnt family members, *Wnt4* and *Wnt2*, which are expressed in the epidermis or the mesoderm and for which mutant alleles exist, exhibit bypass phenotypes. In the *Wnt2*, *Wnt4* double mutant no overshooting occurs (**Table 1**) indicating that these Wnt proteins are unlikely to be involved in LTM guidance and attachment.

### Generation of a *dnt* mutant and establishing its role in LTM attachment site selection

In *Drosophila*, there are two other Ryk proteins in addition to DRL, DRL-2 and DNT (reviewed in [22]). Since *drl* mutants were previously reported to display a partially penetrant muscle bypass phenotype [39], we investigated whether the other two Ryk family members are also required for attachment site selection. *Drl-2* mutants exhibit defects in axon guidance in the antennal lobe [41] and synaptic target specificity at the neuromuscular junction [42] and characterized *dnt* mutant alleles have not been reported. We did not observe any bypass phenotypes in the muscle pattern of *Drl-2^E124^* mutants (**Fig. 1D** **;** **Table 1**). Furthermore, the numbers of LTMs bypassing their normal attachment site does not increase beyond those observed in the *drl* mutant in the *drl*, *Drl-2* double mutant (**Table 1**).

**Figure 2:**
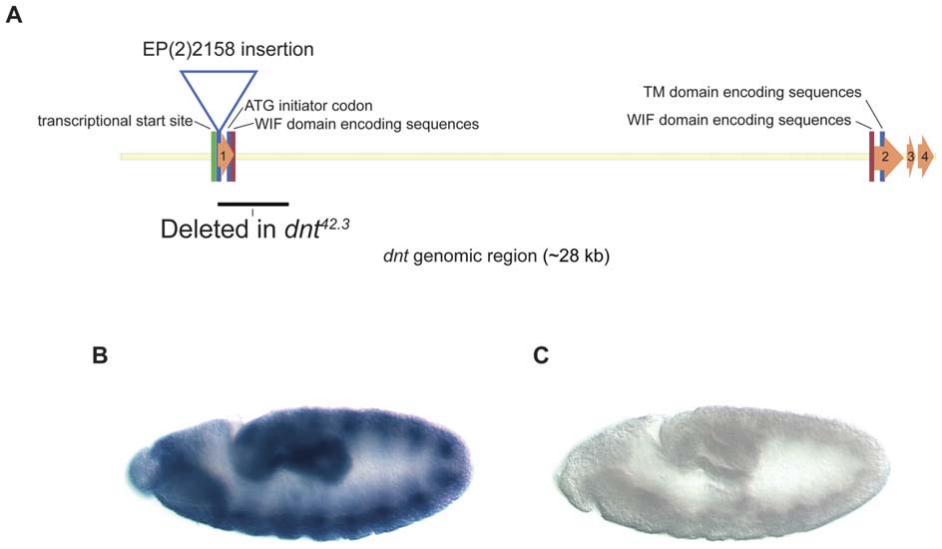
Generation of a *dnt* mutant by imprecise excision of an adjacent P-element. The P{EP}dnt^EP(2)2158^ insert, situated 350 bp upstream of the *dnt* ATG initiator codon, was mobilized by providing a source of transposase and imprecise excisions were selected for by loss of the *w^+^* marker in the P-element insert and molecularly characterized by sequencing cloned genomic PCR products (Materials and Methods). The locations of the insertion, the extent of the deletion in the *dnt^42.3^* allele and gene landmarks, e.g., exons and the location of the WIF encoding segments, are shown in (**A**). The *dnt^42.3^* allele displays dramatically decreased expression of *dnt* mRNA. Stage 11 wild type (**B**) and contemporaneously processed *dnt^42.3^* mutant (**C**) embryos hybridized with a *dnt* antisense probe are shown. Anterior is to the left and dorsal is up in panels (**B**) and (**C**).

We used a P-element mobilization strategy starting with a P-element, P{EP}dnt^EP2158^, inserted 350 base pairs upstream of the *dnt* ATG translational initiator codon to generate mutants in this gene (Materials and Methods). A mutant allele of *dnt*, *dnt^42.3^*, was obtained by imprecise excision of the P-element resulting in a deletion of 2322 base pairs uncovering most of the first exon of the *dnt* transcript. This deletion removes the ATG initiator codon, the first 15 amino acids of the Wnt-binding WIF domain and the 3′ splice donor site (**Fig. 2A**). RNA *in situ* analyses of the *dnt^42.3^* line indicates that the mutant embryos have no detectable *dnt* transcript (compare **Figs. 2B and 2C**). Mutants are viable as homozygotes and analysis of their embryonic musculature indicates that a LTM bypass phenotype is observed in 8% of the hemisegments in the absence of *dnt* (**Table 1**), while the rest of the muscle pattern appears normal.

### WNT5 likely acts through DRL and DNT to mediate muscle attachment site selection

The proximity of the *drl* and *dnt* genes to each other precluded us from recombining a *drl* allele with *dnt^42.3^* to make a mutant line lacking both proteins. Therefore, in order to investigate whether *drl* and *dnt* function redundantly in LTM attachment site selection, we employed two independently generated deficiency chromosomes that uncover both genes. Deficiency Df(2L)ED1231 has breakpoints at 37C5 and 37E3 while the smaller deficiency Df(2L)Exel6043 at 37C5 and 37D7 (http://flybase.org/). Transheterozygotes bearing one copy of either deficiency and one copy of the *drl* mutation show an increase in penetrance of the bypass phenotype to 50% (as compared to 36% in the *drl* homozygous mutant), while *dnt^42.3^/Df(2)Exel6043* embryos have a phenotypic penetrance of 8% (**Table 1**). Virtually all hemisegments display one or more bypassing LTMs (96%) in embryos homozygous for either deficiency (**Fig. 1F**, **Table 1**). These results indicate that *drl* and *dnt* likely act together to mediate appropriate attachment of the LTMs.

We generated animals bearing the Df(2L)Exel6043 deficiency in the *Wnt5* mutant background to determine whether *Wnt5* interacts with *drl* and *dnt*. Neither *Wnt5* nor the deficiency heterozygotes display the muscle bypass phenotype, however females which are heterozygous for *Wnt5*, *drl* and *dnt* display a penetrance of 16% (**Table 1**). Furthermore, males which are hemizygous for *Wnt5* and heterozygous for the deficiency display bypassing muscles in 27% of hemisegments (**Table 1**), a significant increase over the 17% observed in *Wnt5* mutant homozygotes. Thus, we conclude that WNT5 likely signals via both DNT and DRL during muscle attachment site selection.

### LTM ectopic attachments in the *Wnt5* and *drl* mutants are stable and persist through the larval stages

**Figure 3:**
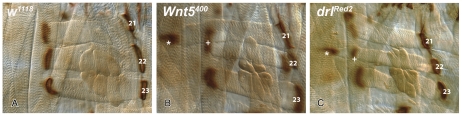
Muscle attachment defects persist from the embryonic to larval stages in *Wnt5* and *drl* mutants. Third instar larval body walls of *w^1118^* (**A**), *Wnt5^400^* (**B**) and *drl^Red2^* (**C**) mutant larvae are stained with anti-FAS2 (mAb 1D4). *Wnt5^400^* larvae and *drl^Red2^* larvae frequently bypass their normal attachment sites and extend ventrally where they form new stable attachments. The original and ectopic tendons cells are indicated by + and *, respectively. FAS2 protein is evident at both sites. The penetrance of the bypass phenotypes is indicated in **Table 1**. Anterior is up and ventral is left.

We next addressed whether the ectopic attachments formed during embryogenesis are maintained to later stages of development. At stage 17 of embryonic development motoneurons innervating the body wall musculature become electrically active, enabling the larvae to use its muscles to push out through the vitelline membrane. The surface area of the larval musculature increases by approximately a 100-fold in the ensuing stages of larval development. We examined LTM attachment in *Wnt5* and *drl* mutants in 3^rd^ instar larvae just before puparation (5 days AEL) by staining the muscle fibers and their attachments with an antibody against the cell adhesion protein FAS2 (Materials and Methods). The larval LTMs were found to frequently extend beyond their normal attachment sites. 8% and 16% of hemisegments contained bypassing LTMs in the *Wnt5* and *drl* mutant larvae, respectively (compare **Fig. 3A** with **Figs. 3B and 3C** **;** **Table 1**). These percentages are roughly half of what is observed at late embryogenesis suggesting that mutant larvae may have decreased survival rates due to defects in the nervous system [28] or other tissues. Clearly, however, a number of the ectopic attachment sites withstand the mechanical stress of hatching and the vigorous locomotion associated with larval feeding behavior.

### Wnt5 protein and mRNA are present in LTMs and in tendon cells

**Figure 4:**
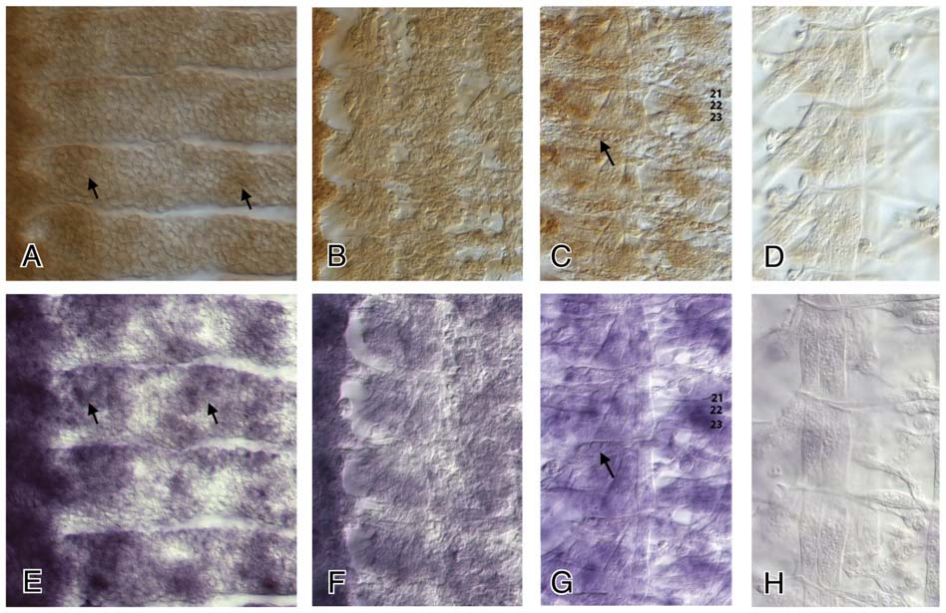
Wnt5 protein and mRNA expression domains in epidermis, muscle and tendon cells during embryonic development. WNT5 is predominantly expressed in subsets of neurons in the CNS from stage 12 onwards throughout embryonic development (data not shown; [28]). However, there is also strong expression from this stage onwards in the epidermis and the musculature. At stage 12, Wnt5 protein (**A, B**) and *Wnt5* mRNA (**E, F**) expression is observed in the epidermis, most prominently in two clusters (**arrows**), and throughout the somatic mesoderm that will give rise to the body wall musculature. Later in embryonic development at early stage 16 WNT5 protein and *Wnt5* mRNA are present in the attachment sites (**arrows in panels C and G**) and at low levels in most muscle fibers including the LTMs 21, 22 and 23 (**C, G**). At the end of embryonic development at late stage 17, Wnt5 protein (**D**) and *Wnt5* mRNA (**H**) are almost undetectable in the somatic mesoderm. In all panels anterior is up and ventral is left.

To examine whether WNT5 is needed in either the muscle or the tendon cells for correct attachment we first determined the developmental patterns of WNT5 expression using anti-WNT5 antisera (Materials and Methods). WNT5 protein expression is first detected at stage 12 (approximately 10 hours AEL) in the CNS [28], throughout the epidermis with some accumulation in ventral and dorsal clusters of cells (**Fig. 4A**) and in the somatic mesoderm (**Fig. 4B**). This is the stage when founder cells fuse with myoblasts and generate the first extending myotubes. WNT5 can be detected in most muscle fibers, including the LTM muscle fibers 21 to 23, and in the tendon cells at early stage 16 when the individual muscle fibers are formed (**Fig. 4C**). WNT5 expression levels are significantly reduced at the end of embryonic development (stage 17) (**Fig. 4D**). A very similar temporal and spatial pattern of expression was observed for *Wnt5* mRNA (**Figs. 4E–H**), suggesting that secreted WNT5 protein is present on or close to the cells in which it is produced. No Wnt5 protein or mRNA was detected in the *Wnt5^400^* mutant embryos (data not shown; [28]).

**Figure 5:**
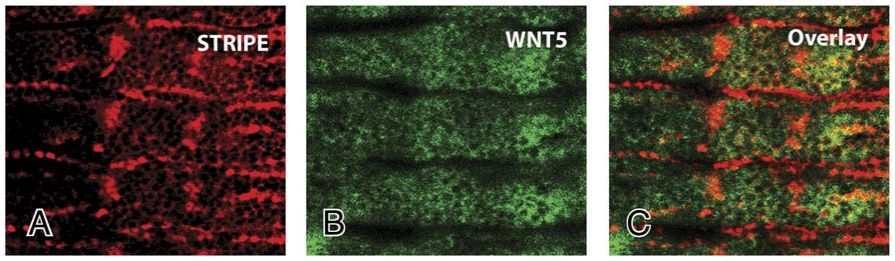
The WNT5 and SR epidermal expression domains partially overlap. Wild type embryos of stage 13 were double labeled with anti-SR and anti-WNT5 antibodies and visualized by confocal microscopy. SR protein (red) is present in a number of tendon precursor cells (**A**). The WNT5 epidermal expression domains (green) in these same embryos are shown in (**B**). The overlay of these panels is shown in (**C**). The SR expression domains partially overlap with the larger WNT5 domains. Anterior is up and ventral is left.

DRL expression in the mesoderm is first detectable around 10 hours AEL and is predominantly concentrated in the developing LTMs and its expression diminishes significantly by stage 16 when the fibers have made their attachments to the tendon cells [39]. DRL is expressed very early in development from 6 hours AEL onwards in reiterated stripes in the epidermis. DRL expression is also observed in clusters that partially co-localize with the SR expression domains at stage 13 [39]. We observe a similar partial co-localization of the SR and WNT5 protein domains in the early tendon cell precursors (**Fig. 5**).

**Figure 6:**
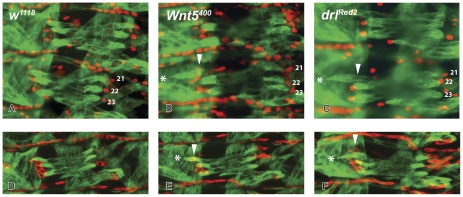
The new attachment sites of the bypassed muscle fibers in *Wnt5* and *drl* mutants frequently do not express SR, while the bypassed attachment sites do. Double labeled stage 16 embryos are shown of *w^1118^* (**A**), *Wnt5^400^* (**B**) and *drl^Red2^* (**C**) with anti-Muscle Myosin in green and anti-SR in red (Material and Methods). Asterisks mark the novel attachment sites of the overshooting LTM muscles; white arrowheads mark the locations of the original attachment sites. In *Wnt5* mutants the novel target sites do not express SR in 65% of the segments containing overshooting muscles, while the bypassed attachment sites usually express SR. The SR positive, original tendon cell is also present in *drl^Red2^* mutants, but is partly masked by the overshooting muscle fiber in panel (**C**), but clearly visible in panel (F)). These results were confirmed in embryos that express Tau-MYC under the control of a *stripe* promoter in both *Wnt5* and *drl* mutants (data not shown). The following genotypes are shown, the control UAS-Tau-MYC; *sr*-GAL4 embryos (**D**), *Wnt5^400^*; UAS-Tau-MYC/*sr*-GAL4 (**E**) and *drl^Red2^*; UAS-Tau-MYC/*sr*-Gal4 (**F**). Anti-Muscle Myosin is shown in green and anti-MYC in red. No MYC protein is observed in the ectopic attachment sites. The photographs in Panels (**A**–**C**) were taken on a compound microscope and those in Panels (**D–F**) on a confocal microscope. Anterior is up and ventral is left.

As development continues SR expression becomes confined to the epidermal tendon cells contacted by a muscle fiber. They are located at the segmental borders for the longitudinal muscles, at ventrally and dorsally located cells for attachment of the ventral and dorsal muscles, and in a few lateral groups where the LTMs attach (**Fig. 6**; [12]).

### Expression of WNT5 in either tendon cells or muscle fibers is sufficient to establish correct LTM attachment

We used the yeast UAS-GAL4 transactivation system ([43]; Materials and Methods) to determine whether WNT5 is required by the approaching myofiber or the tendon cell for correct attachment site selection. We expressed WNT5 specifically in the developing muscle fibers (mef2-GAL4), the tendon cells (sr-GAL4) or both (24B-GAL4) in the *Wnt5* mutant background. mef2-GAL4 drives expression from early mesoderm formation onwards (stage 10) and in the somatic muscle throughout embryonic development [44]. 24B-GAL4 also drives expression in mesoderm and somatic muscle from stage 10 onwards but is also present at the muscle attachment sites [43]. sr-GAL4 expression follows the endogenous expression pattern of the *stripe* promoter and is expressed in tendon cells and its epidermal precursors [45]. We found that expression of WNT5 in all muscle or in the tendon cells or in both, rescues the bypass phenotype in the otherwise *Wnt5* mutant background (**Table 1**). Wnt5 expression in a single ventral longitudinal muscle (muscle 12) that is located in the region into which the bypassed muscle extends, does not inhibit extension of the bypassed muscle fibers (genotype: *Wnt5^400^*; UAS-Wnt5/P{GawB}tey^5053A^, **Table 1**).

We confirmed the earlier report [39] that when DRL is ectopically expressed in muscle only (genotype: *dlr^Red2^*; mef2-GAL4/UAS-*drl*) the bypass phenotype is fully rescued, while no rescue occurs when DRL is expressed only in the attachment sites (genotype: *drl^Red2^*; sr-GAL4/UAS-*drl*) (data not shown). These results indicate that DRL expression is required in the muscle fiber while WNT5 can either be expressed in certain muscles or in the tendon cells to restore correct attachment of LTMs.

### The bypassed tendon cells continue to express the SR protein while the novel attachment sites do not in the majority of the hemisegments

The failure of the LTMs in *drl* and *Wnt5* mutant embryos to recognize their correct attachment sites in the epidermis might be a consequence of alterations in the fate or the formation of the tendon cells. The presence of SR in these cells is both necessary and sufficient for tendon cell fate [11], [13]. Early in development (stage 12/13) the epidermal clusters of tendon cell precursors labeled by SR protein in *Wnt5* and *drl* embryos are similar in size and location to the wild type clusters (data not shown). Later in development, when muscle fibers and tendon cells are fully differentiated, we co-labeled the muscle fiber (with anti-Muscle Myosin) and the tendon cells (with the anti-SR antibody) in *Wnt5* and *drl^Red2^* mutant embryos and again did not observe any apparent obvious changes in SR expression patterns. More specifically, we find that the original bypassed attachment sites continue to express SR (**Figs. 6A–C**). However, the novel epidermal attachment sites that connect the bypassing muscle to the exoskeleton do not express SR in 65% of the hemisegments scored (**Figs. 6A–C**).

**Figure 7:**
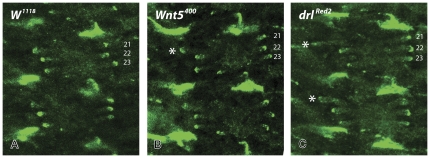
The new attachment sites of the bypassed muscle fibers in *Wnt5* and *drl* mutants express βPS integrin. Wild type (**A**), *Wnt5^400^* (**B**) and *drl^red2^* (**C**) embryos were labelled with anti-βPS Integrin. Muscles 21–23 do exhibit an accumulation of βPS Integrin protein at the tip of the overshooting fibers (white asterix).

We confirmed these results by examining embryos of the genotypes *Wnt5^400^*; UAS-Tau-MYC/sr-GAL4 and *drl^Red2^*; UAS-Tau-MYC/sr-GAL4 for Myc and Muscle Myosin expression (**Figs. 6D–F**). We conclude that the presence of the SR protein in the bypassed tendon cell indicates that overshooting by the muscle fiber is a result of a defect of muscle guidance in *drl* and *Wnt5* mutant embryos, rather than due to alterations in the fate or formation of the appropriate tendon cell. βPS integrin, a protein associated with the myotendinous junction formed at the end of tendon cell determination, accumulates at the tip of the overshooting muscle in the *Wnt5* and *drl* mutant embryos (**Fig. 7**).

## Discussion

The development of the intricate muscle pattern of higher organisms requires the coordinate expression of numerous cellular factors regulating the specific fate, differentiation, orientation and attachment of the individual muscle fibers. The first steps of muscle formation likely occur autonomously, but guidance of myofibers towards and attachment to their appropriate tendon cells are, at least in part, controlled by secreted and transmembrane proteins emanating from both the target cell and the approaching muscle fiber. Here, we have shown that, in *Drosophila*, the secreted WNT5 protein and the Ryk transmembrane receptor family members, DRL and DNT, are essential for guidance of a subset of embryonic body wall muscle fibers to their tendon cells.

There are three Ryk orthologs in *Drosophila*, *drl*, *dnt* and *Drl-2* (reviewed in [22]). We find that 36%, 8%, 0% of hemisegments display a LTM muscle bypass phenotype when *drl*, *dnt* or *Drl-2* is absent, respectively. Homozygosity for relatively small deficiencies that uncover both *drl* and *dnt* results in the bypass phenotype in virtually all hemisegments (96%). Embryos which completely lack DRL and are heterozygous for a mutant allele *of dnt* display intermediate penetrance of the phenotype (50%). Embryos lacking DNT and are heterozygous for *drl* have bypassing muscles in 8% of their hemisegments. These results suggest that the Ryk family members, *dnt* and *drl*, coordinately regulate the attachment of the LTM muscle fibers to tendon cells with *drl* being the dominant player. The decrease in penetrance in the animals lacking both copies of *drl* and one copy of *dnt* (50%), relative to those completely lacking both genes (96%), indicates that *dnt* can at least partially compensate for the absence of *drl*. Consistent with this is the reported ability of the expression of *dnt* in the LTMs to partially rescue the *drl* mutant bypass phenotype [46].

Does WNT5 signal through DNT and DRL? Our genetic studies indicate that this is likely the case. Female embryos simultaneously heterozygous for *Wnt5* and a deficiency which uncovers both *drl* and *dnt* display the bypass phenotype while those heterozygous for either *Wnt5* or the deficiency alone do not. Furthermore, male *Wnt5* mutant hemizygotes, display increased penetrance when single copies of *drl* and *dnt* are removed. Thus, we conclude that *Wnt5* genetically interacts with *drl* and *dnt*, likely indicating that the WNT5 protein acts as a ligand for these two Ryk family members during muscle attachment site selection.

DRL is specifically expressed at the muscle tips of fibers 21–23 while they are in the process of extending towards their attachment sites [39]. The protein is also expressed early in development from 6 hours AEL (stage 10) onwards in reiterated stripes in the epidermis and at stage 12 in clusters of epidermal tendon precursor cells, partially overlapping with the SR expression domain [39]. Rescue of the *drl* mutant LTM bypass phenotype was only achieved when DRL was restored in the muscle and not the attachment sites. At present, the role of the early expression of *drl* in the tendon precursor cells is not clear.

*dnt* mRNA is also expressed in stripes in the epidermis associated with invaginating cells [46], [47]. This transcript is also present at a low level in many embryonic tissues including the somatic musculature. Like DRL, DNT is likely required in the muscle fiber since transgenic expression of *dnt* in the LTMs rescues the *drl* phenotype. DRL-2 is most predominantly expressed in the central nervous system (data not shown), suggesting that it was unlikely to have a role in LTM guidance, as was shown in this study. While almost all hemisegments display overshooting LTMs in the absence of DRL and DNT, only one or two of the three LTM fibers, usually muscles 21 and/or 23, exhibit this phenotype. This result indicates that other non Ryk-dependent mechanisms are required to guide these three muscles to their attachment sites. Alternatively, these two muscles may experience fewer physical barriers blocking their ventral extension beyond muscle 12. In addition, the overshooting of the appropriate tendon cells by these muscles is only observed at the ventral and not the dorsal attachment sites, indicating that guidance mechanisms differ for the two ends of the muscle.

WNT5 has an important role in guidance of embryonic central nervous system commissural axons [28], [29] and the salivary glands [48] and acts as a ligand for DRL in these tissues. When we investigated LTM trajectories in *Wnt5* mutant embryos we found that one or more LTMs overshoot their normal tendon cells in only 17% of the hemisegments compared with 36% in the *drl* mutant. This result suggests that there are likely other DRL ligands in addition to WNT5. Possible other candidates include the other six *wnt* genes present in *Drosophila*, *wg*, *Wnt2*, *Wnt4*, *Wnt6*, *Wnt8* and *Wnt10* (reviewed at “The Wnt Home page” (www.stanford.edu/group/nusselab/cgi-bin/wnt)). Segmentation defects during early embryogenesis in *wg* mutants and the lack of available mutants for *Wnt6* and *Wnt10* precludes further analyses of muscle pattern formation in the absence of these genes. Furthermore, *Wnt8* is not detectably expressed in the somatic mesoderm [49], [50]. Since both *Wnt2* and *Wnt4* had been previously implicated in diverse stages of muscle formation and function [42], [51] we analyzed LTM trajectories in a *Wnt2*/*Wnt4* double mutant. We did not observe any bypassing LTMs in the double mutant embryos, nor in the singly homozygous mutants, indicating that these two Wnt genes are not likely involved in regulating LTM attachment. WNT10 is the most probable alternative ligand for DRL and DNT in muscle since its mRNA is expressed in the developing somatic mesoderm [52], however evaluation of its potential roles awaits the generation of a mutant allele.

In which cells is WNT5 expressed and required? We found that *Wnt5* mRNA and protein are expressed at low levels in all somatic muscles while they are extending, in mature attachment sites and also during early development in a subset of the tendon cell precursors and in the epidermis. Furthermore, rescue of the bypass phenotype is seen when a *Wnt5* transgene is expressed in either of these two tissues. Since WNT5 is a secreted factor and rescue of the *Wnt5* phenotype is observed with restoration in either the muscle or the tendon cells, it is difficult to conclude unambiguously in which tissue it is needed. Restoring expression of WNT5 in muscle fiber 12 only does not rescue the bypass phenotype. This result suggests that it is not simply sufficient to have a high source of WNT5 in the muscle close to the original attachment sites for appropriate inhibition of LTM extension. It is more likely, that WNT5, which is widely expressed in the epidermis and musculature, is modified in some way to become locally activated as a specific LTM repulsive guidance cue. Support for this hypothesis comes from previous observations that Wnt5 is proteolytically-processed [53]. Furthermore, WNT5 expressed by anterior commissural midline glial cells, but not in all neurons, blocks anterior commissure formation [28] due to the repulsion of DRL^+^ axons, indicating that elevated local expression of WNT5 can have different outcomes depending on the cell types which express it. Finally, although WNT5 is observed to be widely expressed in the larval/adult brain, it acts specifically to guide mushroom body α-lobe axons [54] indicating that an apparently ubiquitously-expressed ligand can act as a directional cue. Alternatively, WNT5 may be sequestered from some regions of the extending muscle fiber by so-called “extrinsic receptors” [55] which results in a directional cue received by the leading edge of the muscle.

There is mounting evidence that the final differentiation of the *Drosophila* tendon cell, in particular the secretion of an elaborate extracellular matrix, is tightly coupled to the arrival of the muscle fiber (reviewed in [2]). The resulting myotendinous junction is essential for force transmission and counteraction of muscle contraction by tendon cells. Similar junctions exist in vertebrates where tendons attach the muscles to the bone. In *Drosophila*, it consists of hemi-adherens junction formed by the association of integrin receptor heterodimers on the muscle tip and the tendon cell with the intercalating ECM proteins [2] such as Laminin and TSP secreted from the tendon cells and Tiggrin from the muscle cell. The myotendinous junction is not functional when integrin, TSP or laminin are absent resulting in dissociation of fibers from their attachment sites which leads to lethality. The signals allowing recognition of the appropriate tendon cell, arrest of muscle fiber extension and the formation of the myotendinous junction remain unclear. However, genetic phenotypic analyses indicate that changes in local integrin receptor accumulation on muscle tips and differential responses to TSP presented on the tendons might slow down and stop muscle migration prior to the initiation of myotendinous junction formation (reviewed in [2]). A functional myotendinous junction is formed at the novel attachment site of *Wnt5* and *drl* mutants as evidenced by our observation that βPS integrin accumulates at this site. We do not observe βPS integrin expression at the original attachment site indicating that the interaction of the muscle tip with the bypassed site, if it occurs at all, is not of sufficient duration to initiate attachment site maturation.

The observation that the initial outgrowth and guidance of the LTMs are normal in *Wnt5* and *drl* mutants suggests that these proteins act during the recognition of the target cell and not earlier during muscle extension. Wnt/Ryk signaling may be required for induction of a localized “stop” signal for the LTM at its normal attachment site. In this scenario DRL and DNT present on muscle fibers 21–23 would bind activated WNT5 secreted from their normal attachment sites. This interaction might then result in the transcription of genes encoding extracellular matrix proteins in the muscle fiber which are required to increase adhesiveness between the muscle and tendon cell, slowing down the fibers extension. When either WNT5 or DRL/DNT is absent this signal is not appropriately received by the approaching fiber and it overshoots its target and attaches relatively randomly to a more distant epidermal cell.

In the *Drosophila* embryonic CNS, DRL acts as a repulsive guidance receptor on growth cones of anterior commissural axons to steer them away from the posterior commissural axons which express WNT5. It seemed thus possible that DRL/DNT also acts in the muscle as a repulsive receptor upon binding of WNT5. However, we did not observe any clear muscle guidance defects when WNT5 was ectopically expressed on either specific muscle fibers or in the tendon cells (data not shown). As mentioned above, it is possible that WNT5 has to be locally modified and activated or differentially sequestered to function as a guidance cue in this tissue.

We found that that the novel attachment site for the overshooting muscle in embryos and larvae is an epidermal cell and not another muscle. The normal LTM attachment site that is not recognized by the bypassing muscle is present in *Wnt5* and *drl* mutants as visualized by its ability to express SR, a transcription factor that is both necessary and sufficient to drive tendon cell fate. Therefore, this tendon cell follows important early stages of normal tendon cell differentiation, but does not bind the fiber.

In contrast, only 35% of the ectopic tendon cells express SR suggesting that SR expression is not obligatorily required for formation of a stable myotendinous junction. At present, we do not know whether the novel attachment site expressed SR earlier in development or whether, despite its stability against contraction-induced damage, the ectopic myotendinous junction is different in some manner from the normal junction as to not allow maintenance of SR expression. We find that the FAS2 protein that is normally expressed at the muscle tip and the tendon cell to which it attaches, is present at both the original and the novel attachment sites in *drl* and *Wnt5* mutant larvae. This result indicates that the muscle “filopodia” likely transiently interact with its normal tendon cell target but does not cease extension. This further supports the notion that Wnt/Ryk signaling may increase the stability of muscle/tendon cell interactions.

It is too early to evaluate whether the molecular mechanisms of muscle attachment site selection are conserved between vertebrates and invertebrates because of the paucity of knowledge about the molecules required for tendon differentiation and its connections to muscle and skeletal tissues in vertebrates. Components of Integrin-mediated adhesion complexes, e. g., talin 1 and talin 2 and several laminin integrin receptors were, however, recently shown to be essential for the formation of the vertebrate myotendinous junction [56], as has been observed for their orthologs in *Drosophila* (reviewed in [2]). In the coming years, as more becomes known about the mechanisms that mediate the connections between muscles and tendons, it will be apparent whether other aspects of muscle guidance and target site selection are also conserved.

## Materials and Methods

### Drosophila stocks

All *Drosophila* stocks were grown on standard cornmeal medium at 22°C. The following mutant alleles, GAL4 drivers and UAS-reporter lines were obtained from their originating laboratories or the Bloomington *Drosophila* Stock Center and used in this study: *w^1118^*, *Wnt5^400^* [28], *drl^Red2^* [23], *Drl-2^E124^* [42], *dnt^42.3^* (this study), sr-GAL4 [45], UAS-*Wnt5* [28], 24B-GAL4 [43], mef2-GAL4 ([44], UAS-Tau-MYC [57], P{GawB}tey^5053A^ [58], the *wnt^2L^*, *wnt4^C1^* double mutant and the Df(2L)ED1231 and Df(2L)Exel6043 deficiencies [59].

### Generation of *Dnt* mutants

A mutant allele of *dnt*, *dnt^42.3^*, was generated by imprecise excision of the P-element insertion P{EP}dnt^EP(2)2158^ obtained from the Szeged Stock Center following a standard P-element mobilization strategy [60]. Sequences of the primers used to identify the deletion are available upon request. The *dnt^42.3^* mutant line is viable and has a deletion of 2322 base pairs from positions 19360852 to 19363174 of chromosome 2L (accession number GB:AE014134). This deletion uncovers most of the first exon of the *Dnt* transcript including the ATG initiator codon, the first 15 amino acids of the Wnt-binding WIF domain and the splice donor site. RNA *in situ* analysis of the *dnt* mutant line reveals that the mutant embryos do not detectably express the *dnt* transcript (compare **Figs. 2B and 2C**).

### RNA *in situ* hybridization

Embryo collections for RNA *in situ* hybridization were carried out at 22°C. RNA *in situ* hybridization and staging of embryos were performed as described [28]. *dnt* RNA and *Wnt5* RNA antisense probes were hybridized to paraformaldehyde fixed embryos. The *dnt* probe included positions 386–1225 of the *dnt* RA transcript (accession number NM_057993). The *Wnt5* antisense probe was generated by SP6 polymerase transcription of EcoRV-linearized pOT2-LD22614, which bears the full *Wnt5* open reading frame.

### Immunohistochemistry

All embryo collections for immunohistochemistry were carried out at 22°C. Antibody labelings were performed as described [38]. The following primary antibodies were used on formaldehyde-fixed embryos or third instar larval body walls: anti-Muscle-Myosin mAb (Invitrogen), anti-FAS2 (1D4, Developmental Studies Hybridoma Bank (DSHB)), anti-βPS Integrin (CF.6G11; DSHB), guinea pig anti-SR (gift from T. Volk; [11]), rabbit-anti-GFP (Upstate), anti-Sex-Lethal ([61]; DSHB), rabbit anti-MYC (Upstate) and affinity-purified rabbit anti-WNT5 [28]. Secondary antibodies used were: HRP-conjugated goat anti-mouse or anti-rabbit (Jackson Laboratories) and AlexaFluor-488-conjugated and AlexaFluor-568-conjugated goat anti-mouse and goat anti-rabbit, respectively (Invitrogen). HRP staining was visualized by a standard DAB reaction. After antibody staining, embryos were stored in 70% glycerol in PBS and then dissected and imaged with an Axioplan2 microscope fitted with an Axiocam digital camera (Zeiss) or using an LCS (Leica) confocal microscope.

## References

[1] Schnorrer F, Dickson BJ (2004). Muscle building; mechanisms of myotube guidance and attachment site selection.. Developmental cell.

[2] Schweitzer R, Zelzer E, Volk T (2010). Connecting muscles to tendons: tendons and musculoskeletal development in flies and vertebrates.. Development.

[3] Abmayr SM, Balagopalan L, Galletta BJ, Hong SJ (2003). Cell and molecular biology of myoblast fusion.. International review of cytology.

[4] Abmayr SM, Erickson MS, Bour BA (1995). Embryonic development of the larval body wall musculature of *Drosophila melanogaster*.. Trends in genetics : TIG.

[5] Bate M (1990). The embryonic development of larval muscles in *Drosophila*.. Development.

[6] Bate M, Arias AM (1993).

[7] Volk T (1999). Singling out *Drosophila* tendon cells: a dialogue between two distinct cell types.. Trends in genetics : TIG.

[8] Broadie KS, Bate M (1991). The development of adult muscles in *Drosophila*: ablation of identified muscle precursor cells.. Development.

[9] Haralalka S, Abmayr SM (2010). Myoblast fusion in *Drosophila*.. Experimental cell research.

[10] Piepenburg O, Vorbruggen G, Jackle H (2000). *Drosophila* segment borders result from unilateral repression of hedgehog activity by wingless signaling.. Molecular cell.

[11] Becker S, Pasca G, Strumpf D, Min L, Volk T (1997). Reciprocal signaling between *Drosophila* epidermal muscle attachment cells and their corresponding muscles.. Development.

[12] Volk T, VijayRaghavan K (1994). A central role for epidermal segment border cells in the induction of muscle patterning in the *Drosophila* embryo.. Development.

[13] Vorbruggen G, Jackle H (1997). Epidermal muscle attachment site-specific target gene expression and interference with myotube guidance in response to ectopic stripe expression in the developing *Drosophila* epidermis.. Proceedings of the National Academy of Sciences of the United States of America.

[14] Yarnitzky T, Min L, Volk T (1997). The *Drosophila* neuregulin homolog Vein mediates inductive interactions between myotubes and their epidermal attachment cells.. Genes & development.

[15] Kramer SG, Kidd T, Simpson JH, Goodman CS (2001). Switching repulsion to attraction: changing responses to slit during transition in mesoderm migration.. Science.

[16] Wayburn B, Volk T (2009). LRT, a tendon-specific leucine-rich repeat protein, promotes muscle-tendon targeting through its interaction with Robo.. Development.

[17] Chanana B, Graf R, Koledachkina T, Pflanz R, Vorbruggen G (2007). AlphaPS2 integrin-mediated muscle attachment in *Drosophila* requires the ECM protein Thrombospondin.. Mechanisms of development.

[18] Subramanian A, Wayburn B, Bunch T, Volk T (2007). Thrombospondin-mediated adhesion is essential for the formation of the myotendinous junction in *Drosophila*.. Development.

[19] Dickson BJ, Gilestro GF (2006). Regulation of commissural axon pathfinding by slit and its Robo receptors.. Annual review of cell and developmental biology.

[20] Steigemann P, Molitor A, Fellert S, Jackle H, Vorbruggen G (2004). Heparan sulfate proteoglycan syndecan promotes axonal and myotube guidance by slit/robo signaling.. Current biology : CB.

[21] Schnorrer F, Kalchhauser I, Dickson BJ (2007). The transmembrane protein Kon-tiki couples to Dgrip to mediate myotube targeting in *Drosophila*.. Developmental cell.

[22] Fradkin LG, Dura JM, Noordermeer JN (2010). Ryks: new partners for Wnts in the developing and regenerating nervous system.. Trends in neurosciences.

[23] Bonkowsky JL, Yoshikawa S, O'Keefe DD, Scully AL, Thomas JB (1999). Axon routing across the midline controlled by the *Drosophila* Derailed receptor.. Nature.

[24] Callahan CA, Muralidhar MG, Lundgren SE, Scully AL, Thomas JB (1995). Control of neuronal pathway selection by a *Drosophila* receptor protein-tyrosine kinase family member.. Nature.

[25] Dura JM, Preat T, Tully T (1993). Identification of linotte, a new gene affecting learning and memory in *Drosophila melanogaster*.. Journal of neurogenetics.

[26] Hovens CM, Stacker SA, Andres AC, Harpur AG, Ziemiecki A (1992). RYK, a receptor tyrosine kinase-related molecule with unusual kinase domain motifs.. Proceedings of the National Academy of Sciences of the United States of America.

[27] Stacker SA, Hovens CM, Vitali A, Pritchard MA, Baker E (1993). Molecular cloning and chromosomal localisation of the human homologue of a receptor related to tyrosine kinases (RYK).. Oncogene.

[28] Fradkin LG, van Schie M, Wouda RR, de Jong A, Kamphorst JT (2004). The *Drosophila* Wnt5 protein mediates selective axon fasciculation in the embryonic central nervous system.. Developmental biology.

[29] Yoshikawa S, McKinnon RD, Kokel M, Thomas JB (2003). Wnt-mediated axon guidance via the *Drosophila* Derailed receptor.. Nature.

[30] Logan CY, Nusse R (2004). The Wnt signaling pathway in development and disease.. Annual review of cell and developmental biology.

[31] Bovolenta P, Rodriguez J, Esteve P (2006). Frizzled/RYK mediated signalling in axon guidance.. Development.

[32] Salinas PC, Zou Y (2008). Wnt signaling in neural circuit assembly.. Annual Review of Neuroscience.

[33] Clevers H (2006). Wnt/beta-catenin signaling in development and disease.. Cell.

[34] Kuhl M, Sheldahl LC, Park M, Miller JR, Moon RT (2000). The Wnt/Ca2+ pathway: a new vertebrate Wnt signaling pathway takes shape.. Trends in genetics : TIG.

[35] Simons M, Mlodzik M (2008). Planar cell polarity signaling: from fly development to human disease.. Annual review of genetics.

[36] Manning G, Whyte DB, Martinez R, Hunter T, Sudarsanam S (2002). The protein kinase complement of the human genome.. Science.

[37] Lyu J, Yamamoto V, Lu W (2008). Cleavage of the Wnt receptor Ryk regulates neuronal differentiation during cortical neurogenesis.. Developmental cell.

[38] Wouda RR, Bansraj MR, de Jong AW, Noordermeer JN, Fradkin LG (2008). Src family kinases are required for WNT5 signaling through the Derailed/RYK receptor in the *Drosophila* embryonic central nervous system.. Development.

[39] Callahan CA, Bonkovsky JL, Scully AL, Thomas JB (1996). derailed is required for muscle attachment site selection in *Drosophila*.. Development.

[40] Yoshikawa S, Bonkowsky JL, Kokel M, Shyn S, Thomas JB (2001). The derailed guidance receptor does not require kinase activity in vivo.. The Journal of neuroscience : the official journal of the Society for Neuroscience.

[41] Sakurai M, Aoki T, Yoshikawa S, Santschi LA, Saito H (2009). Differentially expressed Drl and Drl-2 play opposing roles in Wnt5 signaling during *Drosophila* olfactory system development.. The Journal of neuroscience : the official journal of the Society for Neuroscience.

[42] Inaki M, Yoshikawa S, Thomas JB, Aburatani H, Nose A (2007). Wnt4 is a local repulsive cue that determines synaptic target specificity.. Current biology : CB.

[43] Brand AH, Perrimon N (1993). Targeted gene expression as a means of altering cell fates and generating dominant phenotypes.. Development.

[44] Ranganayakulu G, Schulz RA, Olson EN (1996). Wingless signaling induces nautilus expression in the ventral mesoderm of the *Drosophila* embryo.. Developmental biology.

[45] Ghazi A, Anant S, VijayRaghavan K (2000). Apterous mediates development of direct flight muscles autonomously and indirect flight muscles through epidermal cues.. Development.

[46] Oates AC, Bonkovsky JL, Irvine DV, Kelly LE, Thomas JB (1998). Embryonic expression and activity of doughnut, a second RYK homolog in *Drosophila*.. Mechanisms of development.

[47] Savant-Bhonsale S, Friese M, McCoon P, Montell DJ (1999). A *Drosophila* derailed homolog, doughnut, expressed in invaginating cells during embryogenesis.. Gene.

[48] Harris KE, Beckendorf SK (2007). Different Wnt signals act through the Frizzled and RYK receptors during *Drosophila* salivary gland migration.. Development.

[49] Gordon MD, Dionne MS, Schneider DS, Nusse R (2005). WntD is a feedback inhibitor of Dorsal/NF-kappaB in *Drosophila* development and immunity.. Nature.

[50] Ganguly A, Jiang J, Ip YT (2005). *Drosophila* WntD is a target and an inhibitor of the Dorsal/Twist/Snail network in the gastrulating embryo.. Development.

[51] Kozopas KM, Nusse R (2002). Direct flight muscles in *Drosophila* develop from cells with characteristics of founders and depend on DWnt-2 for their correct patterning.. Developmental biology.

[52] Janson K, Cohen ED, Wilder EL (2001). Expression of DWnt6, DWnt10, and DFz4 during *Drosophila* development.. Mechanisms of development.

[53] Fradkin LG, Noordermeer JN, Nusse R (1995). The *Drosophila* Wnt protein DWnt-3 is a secreted glycoprotein localized on the axon tracts of the embryonic CNS.. Developmental biology.

[54] Shimizu K, Sato M, Tabata T (2011). The Wnt5/planar cell polarity pathway regulates axonal development of the *Drosophila* mushroom body neuron.. The Journal of neuroscience : the official journal of the Society for Neuroscience.

[55] Grillenzoni N, Flandre A, Lasbleiz C, Dura JM (2007). Respective roles of the DRL receptor and its ligand WNT5 in *Drosophila* mushroom body development.. Development.

[56] Conti FJ, Monkley SJ, Wood MR, Critchley DR, Muller U (2009). Talin 1 and 2 are required for myoblast fusion, sarcomere assembly and the maintenance of myotendinous junctions.. Development.

[57] Thor S, Thomas JB (1997). The *Drosophila* islet gene governs axon pathfinding and neurotransmitter identity.. Neuron.

[58] Ritzenthaler S, Suzuki E, Chiba A (2000). Postsynaptic filopodia in muscle cells interact with innervating motoneuron axons.. Nature neuroscience.

[59] Ryder E, Ashburner M, Bautista-Llacer R, Drummond J, Webster J (2007). The DrosDel deletion collection: a *Drosophila* genomewide chromosomal deficiency resource.. Genetics.

[60] Tower J, Karpen GH, Craig N, Spradling AC (1993). Preferential transposition of *Drosophila* P elements to nearby chromosomal sites.. Genetics.

[61] Bopp D, Bell LR, Cline TW, Schedl P (1991). Developmental distribution of female-specific Sex-lethal proteins in *Drosophila* melanogaster.. Genes & development.

